# A 24-hour dietary recall for assessing the intake pattern of choline among Bangladeshi pregnant women at their third trimester of pregnancy

**DOI:** 10.5195/cajgh.2014.72

**Published:** 2014-04-17

**Authors:** Shatabdi Goon, Sima Rani Dey

**Affiliations:** 1 Dept. of Nutrition and Food Engineering, Daffodil International University, Dhaka, Bangladesh; 2 Dept. of Applied Statistics, East West University, Dhaka, Bangladesh

**Keywords:** choline, pregnancy, fetal development, cognition, pregnancy outcome

## Abstract

Maternal choline intake during the third trimester of human pregnancy can modify systemic and local epigenetic marks in fetal-derived tissues, promoting better pregnancy outcomes, increased immunity, as well as improved mental and physical work capacity with proper memory and cognitive development. 103 pregnant women presenting to the antenatal care of Azimpur Maternity Hospital of Dhaka, Bangladesh in their third trimester of pregnancy were randomly selected for this cross sectional study exploring dietary intake patterns of choline. A dietary recall form was administered to estimate frequency and amount of food consumption of foods for the previous 24 hours. Most women reported diets that delivered less than the recommended choline intake (mean ± SD; 189.5 ± 98.2) providing only 42.72% of total RDA value. The results of this study may indicate that dietary choline among pregnant, Bangladeshi women may not be adequate to meet the needs of both, the mother and fetus. Further studies are warranted to determine clinical implications.

## Introduction

Choline, an essential 1–6 nutrient found in eggs, liver, milk, meat, nuts, legumes, and cruciferous vegetables, 7 plays a significant role during the third trimester of human pregnancy to reduce the negative effect of a mother’s stress 8 on child health 8,promoting fetal growth, 6,9,10 proper brain 11–16 and memory function, 17–22 and learning capabilities, 23–27 while protecting the future health of the child. Previous findings suggest that higher maternal choline intake may counter some adverse effects 25,28,29 of prenatal stress on behavioral, 29–32 neuroendocrine, and metabolic development 33,34 in offspring. Higher choline intake contributed to a more stable Hypothalamic-Pituitary-Adrenal (HPA) axis, 8 which translated to lower cortisol levels in the fetus. Changes in fetal genetic expression likely continue into adulthood, where they play a role in stress-related disease prevention. 35 Dietary choline intake by the pregnant mother and by the infant directly affects brain development and results in permanent changes in brain function. 36 Variations in maternal choline intake influence memory performance in their offspring. 37

Several animal model studies have reported on the effect of choline intake and fetal development. Offspring born to pregnant rats given choline supplements were found to be faster learners with better memories. 12 More choline during days 11 to 18 of gestation resulted in increased cell proliferation and decreased apoptosis in rodent fetal hippocampal progenitor cells, promoting better memory and cognitive function. 12 Choline deprivation (CD)-induced dysfunction in brain mitochondria may be responsible for impairment in cognition and underlines that the brain needs an adequate choline supply for its normal functioning. Memory can be permanently enhanced by exposure to choline during the latter part of gestation. 16 Also, Zeisel *et al*. showed that when rat pups received choline supplements, their brain function changed, resulting in lifelong memory enhancement. 27 Mellot *et al*. showed that increased dietary intake of choline early in life improves performance of adult rats on memory tasks and prevents their age-related memory decline. 22 Choline supplementation during gestation in rats leads to augmentation of spatial memory in adulthood. 38 Maternal choline appears to decrease the risk of neural tube defect (NTD). 39,40 A retrospective case-control study of periconceptional dietary choline intake in California, USA women found that women in the lowest quartile for daily choline intake had a 4-fold greater risk of having a baby with an NTD than women in the highest quartile for intake. 40

Another study showed that a deficiency of choline substantially impaired the body’s ability to regulate homocysteine levels. 41 Excessive homocysteine is apparently linked with increased risks for birth defects, cardiovascular disease, 42 cancer, type-2 diabetes, hypertension, depression, and more. Higher intakes of dietary choline are related to lower homocysteine concentrations. 43,44

Foods rich in choline may help reduce the risk of inflammation associated with chronic diseases such as cardiovascular disease, bone loss, dementia, and Alzheimer’s disease. 43 A study funded by the National Institutes of Health concluded that dietary choline during pregnancy is associated with a 24% reduced risk of breast cancer in female offspring. 45,46 Tumor growth rate was inversely related to choline content in the prenatal diet, resulting in 50% longer survival. Choline deficiency during pregnancy may lead to increased risk of complications during delivery, including prolonged labour, preterm delivery, preeclampsia, prematurity, very low birth weight, 47 and maternal and neonatal death. It is recommended that pregnant women take 450mg of choline per day from common food sources or supplements.

In Bangladesh, knowledge of the impact of maternal food and micronutrient supplementation on infant micronutrient status is limited. Most pregnant Bangladeshi women do not meet recommended levels of micronutrients, including choline. The main objective of this study was to determine the present status of the choline intake pattern by Bangladeshi pregnant women in their third trimester of pregnancy.

## Methods

This cross sectional study was carried out from the 4-May to 6-June, 2013 at Azimpur maternity hospital, Dhaka, Bangladesh. A total of two hundred pregnant women were randomly selected from all pregnant women present the first day of the survey. Of the initial 200, 103 women were in their third trimester of pregnancy and were included in this investigation. Exclusion critieria included: women not in their third trimester of pregnancy, history of hypertension, gestational diabetes, or history of spontaneous abortions. All participants signed an informed consent form.

A semi-structured pre-tested questionnaire (see Appendix 1) was developed to gather participant characteristics including: age, occupation, stage of pregnancy, weight, height, and educational level. A dietary recall form was administrated to gather information regarding dietary intake for the previous 24 hours. Choline content of foods was calculated using published data from the USDA-Nutrient Database for Standard Reference then multiplying the frequency of consumption of each food item by its choline content and summing the nutrient contributions of all foods. All of the collected data were analyzed using SPSS v-15.0. Descriptive statistics including mean, standard deviation, and frequency were obtained. All variables were normally distributed.

## Result

### Subject profile

In the maternity hospital in Dhaka, Bangladesh, 69.9% of participants aged 21–25 years. 27.2% and 2.9% of were aged 26–30 years and >31 years, respectively. Approximately 6.8% of participants were illiterate; 10.7%, 56.3%, 11.7%, and 14.5% completed primary, secondary, undergraduate, and graduate levels of study, respectively. 87.4% of participants were housewives and 12.6% were service holders. In this study, 30.1% of participants were in the seventh month of pregnancy, 28.16% in the eighth month of pregnancy, and 41.74%, the ninth. Based on calculated body mass index (BMI), 3.8% were underweight, 42.72% normal weight, 33.98% overweight, 13.59% moderately obese, 3.88% severely obese, and 1.95% were very severely obese. Table 1 shows the overall subject profile attending the study.

### Dietary choline consumption

Most participants reported low consumption of choline-rich foods selected from the Bangladeshi diet. Only one participant showed adequate or near adequate intake of choline (> 400mg). The mean dietary choline consumed was 189.5mg ± 98.2 (mean ± SD). 25.2%, 31.2%, 23.3%, and 19.4% of pregnant women took choline ranges from 0–100, 101–200, 201–300, and 301–400 mg/day, respectively, through regular diet.

Parallel improvements were observed in average choline intake with educational achievement. Average intake level of choline was 147.7 ± 83.3, 157.8 ± 110.5, 185.9 ± 97.4, 208.2 ± 94.2, and 231.4 ± 94.9 mg/day for illiterate participants, those who completed primary, secondary, undergraduate, and graduate levels of study, respectively. Table 3 illustrates dietary choline consumption.

Among the factors affecting choline level intake, education level and age of participant have substantial effect. Both factors are positively correlated with choline level intake, though the correlation is weak.

## Discussion

Improving choline intake through regular diet benefits all individuals through increasing immunity and lower morbidity from infectious diseases, improving physical work capacity, memory and cognitive development. Pregnant women are less likely to have poor pregnancy outcomes (including perinatal mortality) and may deliver infants with larger birth weights and greater choline stores.

In this study, an assessment of dietary choline intake was made using a dietary recall system to record food intake by pregnant women over the previous 24 hours. Analysis revealed that the choline status of Bangladeshi pregnant women is far below clinical suggestions, as defined by the Institute of Medicine of the National Academy of Sciences (450 mg/day).

Data suggest that pregnant Bangladeshi women are consuming less than adequate amounts of choline, with mean consumption of 189.5 ± 98.2 mg/day. For comparision, in a New Zealand study, 49 daily intake of choline was 316 (± 66) mg/day; in another, mean intake was 304 mg/day in women; 50 in a third study, mean intake of choline by common food sources among a Taiwanese female population 51 was estimated as 265 ± 9 mg/day. A study conducted in Jamaica also showed poor choline status among pregnant women with 278.5 mg/day, which was higher than the Bangladeshi scenario. 52

Bangladeshi pregnant women took only 42.72% of the RDA value of 450mg/day. Poverty and lack of knowledge regarding the importance of choline among both pregnant women and health care professionals leads to less choline supplementation during pregnancy. Therefore, the dietary intake pattern of choline during third trimester of pregnancy is important to estimate.

Maternal age is an important determinant of nutritional status for pregnant women. The ideal age of pregnancy is 19–30 years. In this population, the majority were 20–25 years of age. Maternal education level has a significant effect on choline status during pregnancy. The present study showed a direct relationship between increase in consumption of choline in pregnancy and increase in maternal education level. This may due to generally better understanding of the mother regarding public health knowledge and nutritional status. Weight status also reflects the nutritional status of women. About 43% of pregnant women were categorized as “healthy weight”. 13.59%, 3.88%, and 1.95% of participants were moderately, severely, and very severely obese, indicating an increase level of obesity during pregnancy. The overall survey result also shows that most women don’t consume the recommended level of choline.

This is the first documented study evaluating dietary choline intake among the Bangladeshi population and suggests a need to further assess whether the diets of this population ensure an adequate plasma choline levels during pregnancy. An extension of this study should also examine the implications of low plasma choline concentration including the significance it may have in ensuring healthy fetal brain development in humans.

Our study has a number of limitations. The data was self-reported and the study is cross-sectional which does not infer causal relationships. Furthermore, we examined only one maternity hospital located in Dhaka, Bangladesh. Caution should be taken to generalize the data for other maternity hospitals outside Dhaka city. A 24-hour dietary recall was taken to calculate the daily choline intake. Twenty-four hour recall is a retrospective method of diet assessment, where an individual is interviewed about their food and beverage consumption during the previous day or the preceding 24 hours. However, a single 24-hour recall is not considered to be representative of habitual diet at an individual level. While there may have been a small amount of recall bias, this methodology is adequate for surveying intake in a large group and estimating group mean intakes of diet.

## Conclusion

Choline is a mostly neglected micronutrient by both pregnant women and health care professionals. These professions have poor knowledge regarding its importance and therefore are missing many potential causes of health complications due to choline deficiency. The results of this study may be an indication that the choline included in the diet of pregnant Bangladeshi women may not be adequate to meet both the needs of the mother and fetus. The results presented here may be useful in understanding the present choline status among Bangladeshi pregnant women aiming to improve using strategic nutritional intervention by both government and public stakeholders.

## Figures and Tables

**Table 1:** Characteristics of pregnant women

Variable	n (%)
**Age (years)**	
21–25	72 (69.9%)
26–30	28(27.2%)
> 31	3(2.9%)
**Years of Education**	
Illiterate	7(6.8%)
Primary (Level 1–5)	11(10.7%)
Secondary (Level 6–10)	58(56.3%)
Undergraduate (Level 11–12)	12(11.7%)
Graduate (more than 12 years of education)	15(14.5%)
**Status of Pregnant Women**	
Housewife	90(87.4%)
Service holder	13(12.6%)
**Stage of Pregnancy**	
7th month of pregnancy	31(30.1%)
8^th^ month of pregnancy	29(28.16%)
9^th^ month of pregnancy	43(41.74%)
**BMI Status**	
15–16	0
16–18.5	4(3.9%)
18.5–25	44(42.72%)
25–30	35(33.98%)
30–35	14(14%
35–40	4(3.9%)
Over 40	2

**Table 2:** Distribution of pregnant women by daily intake of choline (mg)

Choline intake level (mg/day)	n (%)
0–100	26 (25.2%)
101–200	32 (31.2%)
201–300	24 (23.3%)
301–400	20 (19.4%)
≥ 400	1 (0.9%)
Total	103

**Table 3:** Gradual improvement of average choline intake per day with educational status

Educational level	Average choline intake/day (mg)
Illiterate	147.7 ± 83.3
Primary	157.8 ± 110.5
Secondary	185.9 ± 97.4
Undergraduate	208.2 ± 94.2
≥ Graduate	231.4 ± 94.9

**Table 4:** Correlation Analysis

Correlations			
	Choline Level	Education Level	Age
Choline Level	1	0.186	0.166
Education Level	0.186	1	−0.008
Age	0.166	−0.008	1
